# Supplementary material for: GPCR signaling regulates severe stress‐induced organismic death in Caenorhabditis elegans
Source: Aging Cell. 2022 Nov 22;22(1):e13735. doi: 10.1111/acel.13735 (PMC9835589; doi:10.1111/acel.13735)
Supplement: Supplementary file 1 — Figures S1‐S4 [file ACEL-22-e13735-s002.pdf]

Figure S1

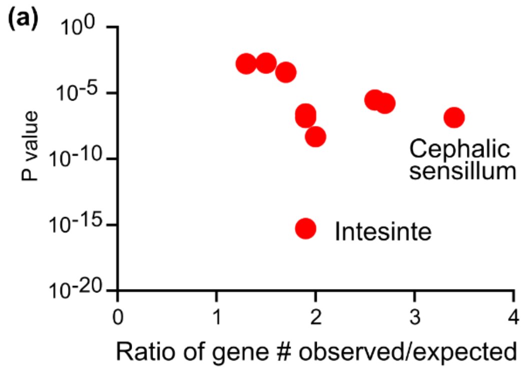

Tissue Enrichment Analysis (TEA) for genes up-regulated by freeze-thaw stress:

Term	Expected	Observed	Enrichment	P value
intestine WBbt:0005772	95	178	1.9	5.3e-16
head mesodermal cell WBbt:0004697	43	84	2	4.9e-09
cephalic sensillum WBbt:0006920	6.7	23	3.4	1.4e-07
PVD WBbt:0006831	39	74	1.9	1.4e-07
outer labial sensillum WBbt:0005501	40	74	1.9	2.7e-07
cephalic sheath cell WBbt:0008406	10	27	2.7	1.7e-06
anterior ganglion WBbt:0005375	10	27	2.6	3e-06
ASE WBbt:0005663	25	43	1.7	0.00037
muscular system WBbt:0005737	95	123	1.3	0.0017
thermosensory neuron WBbt:0005838	31	47	1.5	0.0019

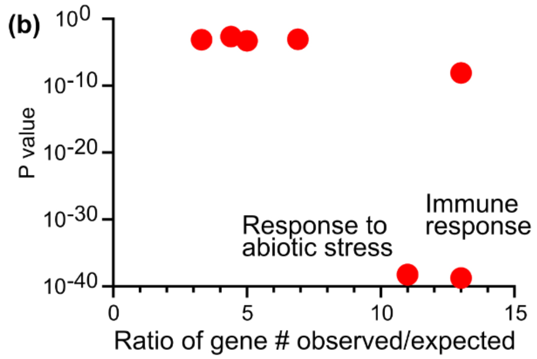

Gene Ontology Enrichment Analysis (GOEA) for genes up-regulated by freeze-thaw stress:

Term	Expected	Observed	Enrichment	P value
immune system process GO:0002376	3.7	48	13	1.8e-39
response to biotic stimulus GO:0009607	4.8	53	11	5.2e-39
interspecies interaction GO:0044419	4.9	53	11	6e-39
defense response GO:0006952	4.9	53	11	7.8e-39
membrane microdomain GO:0098857	0.6	8	13	8.4e-09
transmembrane transporter GO:0015399	1	5	5	0.00052
protein folding GO:0035966	2.4	8	3.3	0.00074
response to axon injury GO:0048678	0.43	3	6.9	0.00091
extracellular stimulus GO:0009991	0.92	4	4.4	0.0023

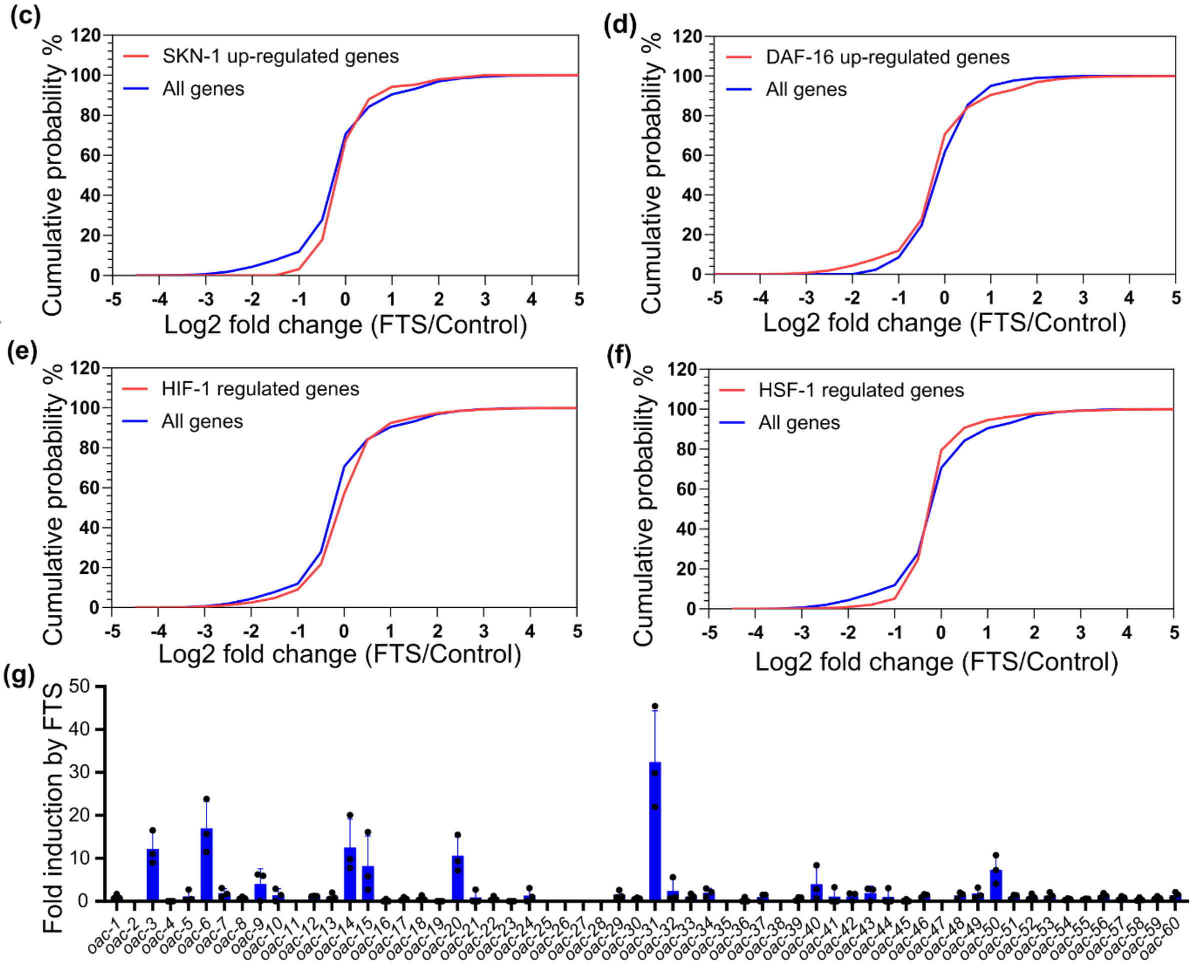

Figure S1: Transcriptome profiling and analysis of genes up-regulated by FTS. **(a)**, Tissue Enrichment Analysis (TEA) of genes up-regulated by FTS showing intestine as a major site of gene regulation. **(b)**, Gene Ontology Enrichment (GOE) Analysis of genes up-regulated by FTS showing abiotic stress and immune response as major processes involved. **(c-f)**, Cumulative probability analysis of genes regulated by SKN-1 (**c**), DAF-16 (**d**), HIF-1 (**e**), HSF-1 (**f**) (Brunquell et al. 2016, p.1; Kumar et al. 2015; Oliveira et al. 2009; Steinbaugh et al. 2015) does not show marked enrichment of those also regulated by FTS (NCBI SRA BioProject accession PRJNA763790). **(g)**, Normalized fold induction of gene expression for the entire *oac* gene family based on RNAseq results, showing *oac-31* as the most up-regulated family member.

Figure S2

(a)

**Signal transduction pathways** (Cytoplasmic membrane proteins transcription factors)	**Genes tested for RNAi phenotype in *oac-31*p::GFP** (Selected with intestinal TPM expression > 2.0) (Blue: baseline GFP expression increased) (Red: freeze-thaw stress induction decreased)
Neuropeptide GPCRs	*npr-2, npr-8, npr-21, npr-25, npr-30*
Wnt receptors	*mom-5, mig-1, cam-1, lin-18*
Shh receptor-like proteins	*ptc-1, ptr-2, ptr-5, ptr-17, ptr-18, ptr-21*
TGF/BMP receptors	*daf-1*
TRP channels	*trpa-1, trpa-2, trp-1, trpl-5, gon-2, cup-5, gtl-1, gtl-2*
Voltage-gated calcium channels	*egl-19, unc-2, nca-2*
Cyclic nucleotide-gated ion channels	*cng-1*
Mechanosensory ion channels	*pezo-1, flr-1*
Innate immune receptors	*octr-1, fshr-1, tol-1*
Progestin/AdipoQ receptors	*paqr-1, paqr-2*
Receptor tyrosine kinases (Insulin, Eph, EGF, FGF receptors)	*daf-2, vab-1, let-23, kin-15*
Transcription factors	298 genes tested including: *hif-1, hsf-1, daf-16, skn-1, sbp-1, tra-1, zip-2, zip-10*

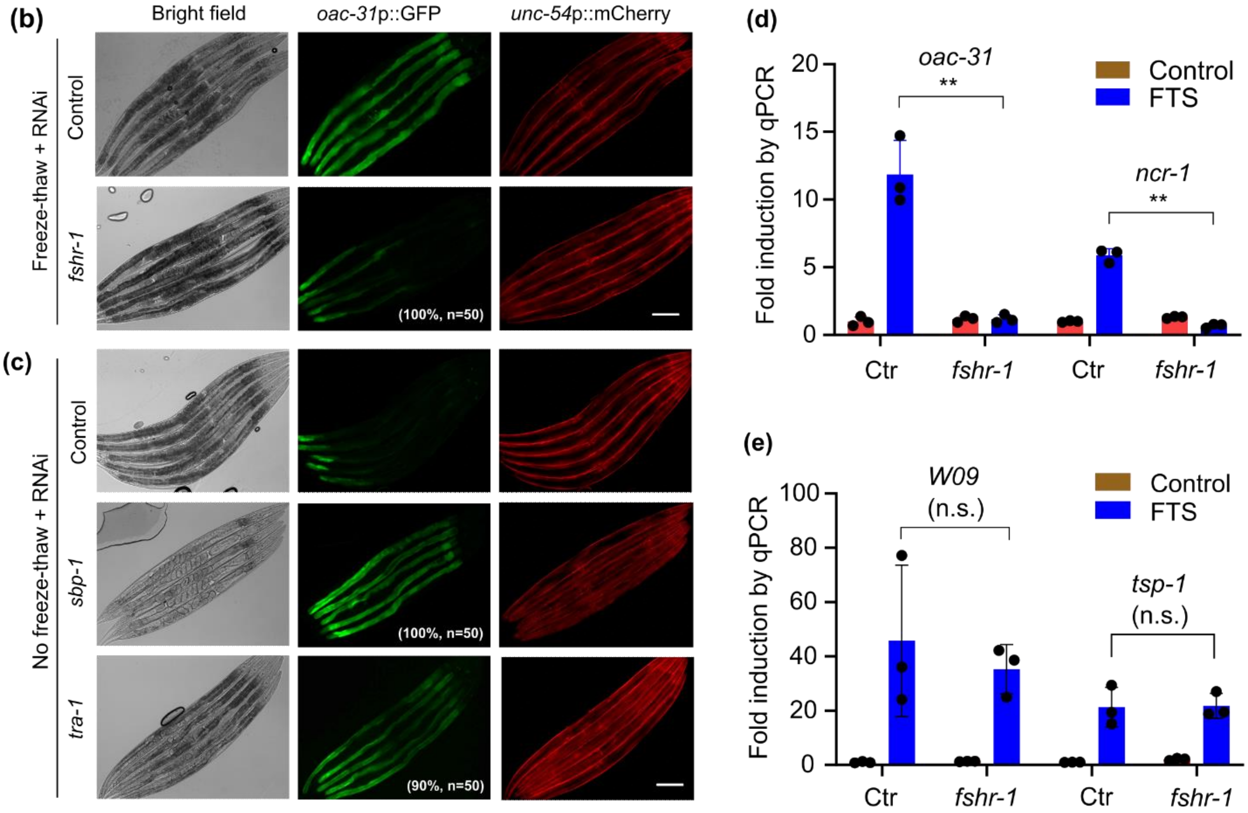

Figure S2: RNAi screens identify genes that regulate *oac-31*p::GFP induction by FTS. (a), Table listing RNAi clones screened for *oac-31*p::GFP induction phenotype. RNAi cloned were selected based on intestinal expression and genes encoding transmembrane receptors and representing major signal transduction pathways. **(b)**, Representative fluorescence images showing suppression of FTS-induced *oac-31*p::GFP by RNAi against *fshr-1*. **(c)**, Representative fluorescence images showing constitutive activation of *oac-31*p::GFP by RNAi against *tra-1* or *sbp-1* in the absence of FTS treatment. Scale bar, 100 µm. **(d),** qRT-PCR results showing abolished induction of *ncr-1*, *oac-31* by FTS in animals deficient in *fshr-1*. **(e),** qRT-PCR results showing normal induction of the genes *W09G12.7* and *tsp-1*, by FTS in *fshr-1* mutants. Values are means ± S.D with $^{**}P < 0.01$ and $^{*}P < 0.05$ (two-way ANOVA for genotype-effect interaction and post-hoc Tukey HSD, N = 3 independent experiments, n > 50 for each experiment). n.s., non-significant. Scale bar, 100 µm.

Figure S3

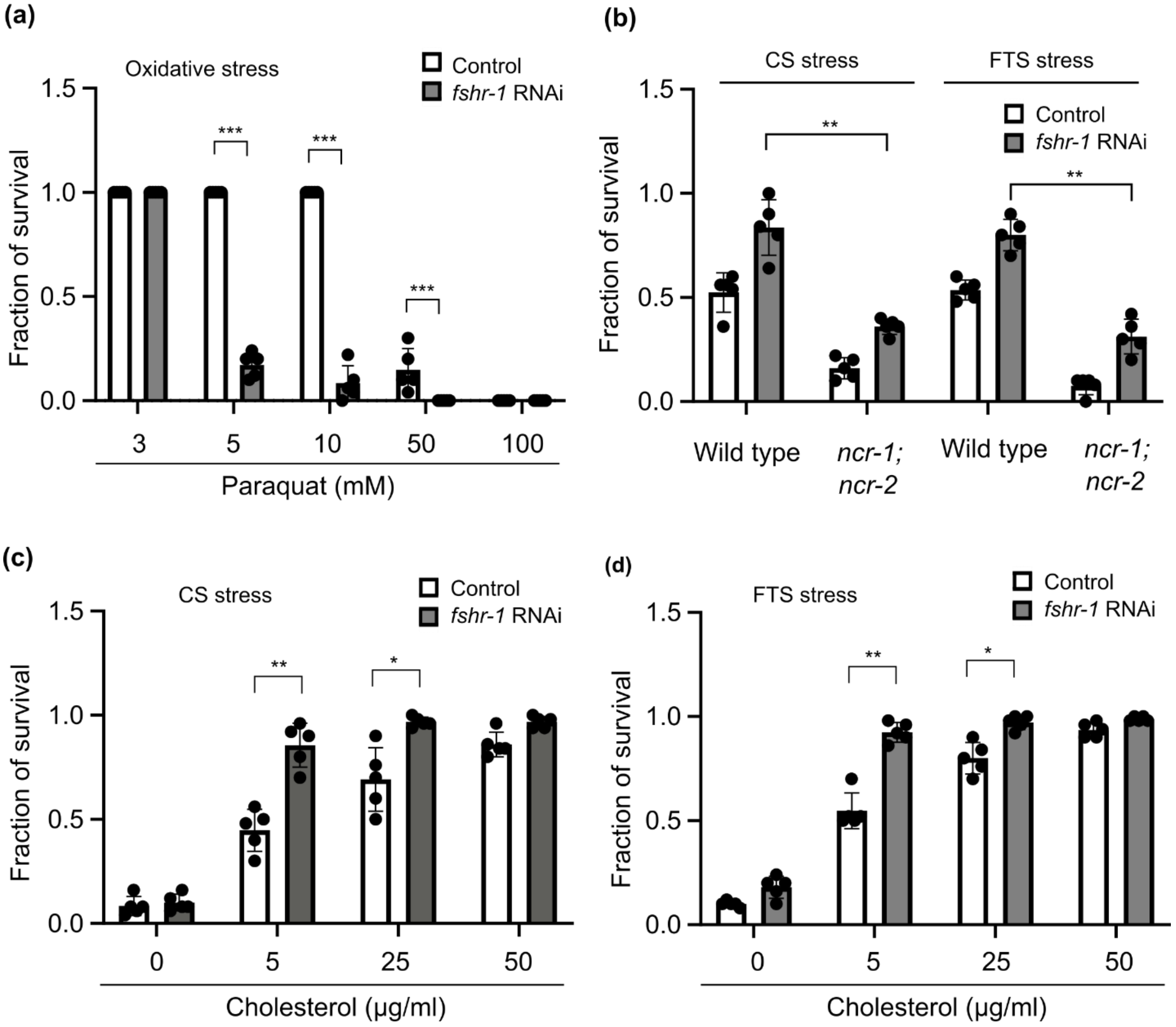

Figure S3: FSHR-1 regulates trade-off of organismic resilience to cholesterol oxidative and hypothermic stresses. (a), Survival rates of control and *fshr-1* RNAi-treated animals grown on increasing doses of Paraquat (3, 5, 10, 50 and 100 mM in NGM). **(b),** Survival rates of wild type or *ncr-1; ncr-2* double mutant animals treated with control or *fshr-1* RNAi after severe FTS (-20 ^{o}C for 45 minutes, followed by recovery for 24 hrs at 25 ^{o}C) or cold shock (CS, 4 ^{o}C for 24 hrs, followed by recovery for 24 hrs at 25 ^{o}C) grown on cholesterol (5 µg/ml). **(c),** Survival rates of control and *fshr-1* RNAi-treated

animals after severe CS grown on increasing doses of cholesterol (0, 5, 25, 50 µg/ml). **(d),** Survival rates of control and *fshr-1* RNAi-treated animals after severe FTS grown on increasing doses of cholesterol (0, 5, 25, 50 µg/ml). Values are means ± S.D with ****P* < 0.001, ***P* < 0.01 and **P* < 0.05 (two-way ANOVA for genotype-effect interaction and post-hoc Tukey HSD, N = 3 independent experiments, n > 50 for each experiment).

Figure S4

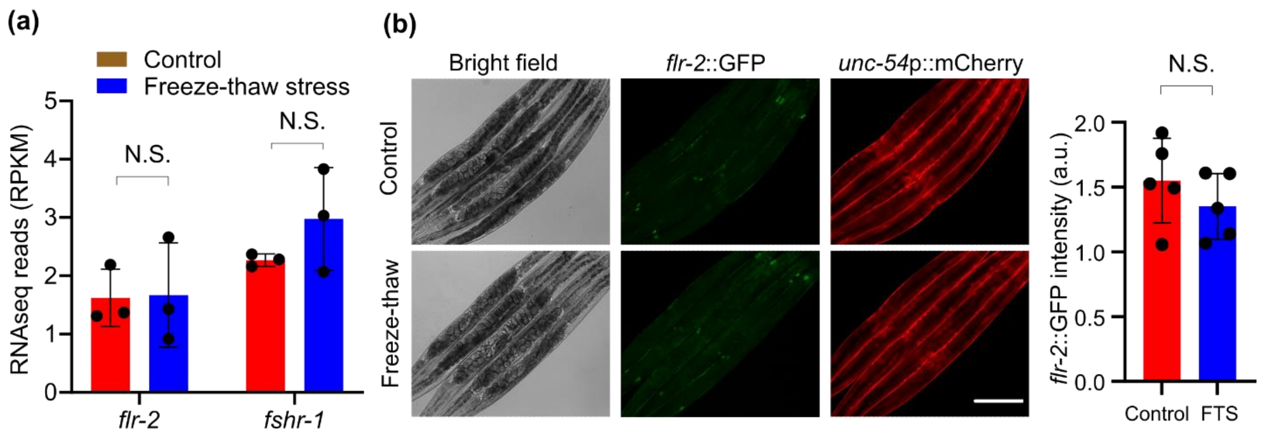

Figure S4: *flr-2*, which encodes the putative ligand of FSHR-1, is essential for FTS induction of *oac-31* but not transcriptionally regulated by FTS. (a), RNAseq results showing no apparent up-regulation of *flr-2* or *fshr-1* by FTS (two-way ANOVA for genotype-effect interaction and post-hoc Tukey HSD, N = 3 independent experiments, n > 50 for each experiment). N.S., non-significant. **(b)**, Representative fluorescence images for the translational reporter *flr-2*p::*flr-2*::GFP showing its normal secretion and enrichment in coelomocytes but without apparent regulation by FTS (unpaired student-t test, N.S., non-significant).

REFERENCES

Brunquell J, Morris S, Lu Y, Cheng F & Westerheide SD (2016) The genome-wide role of HSF-1 in the regulation of gene expression in Caenorhabditis elegans. *BMC Genomics* 17. Available at: http://www.ncbi.nlm.nih.gov/pmc/articles/PMC4975890/.

Kumar N, Jain V, Singh A, Jagtap U, Verma S & Mukhopadhyay A (2015) Genome-wide endogenous DAF-16/FOXO recruitment dynamics during lowered insulin signalling in C. elegans. *Oncotarget* 6, 41418–41433.

Oliveira RP, Porter Abate J, Dilks K, Landis J, Ashraf J, Murphy CT & Blackwell TK (2009) Condition-adapted stress and longevity gene regulation by Caenorhabditis elegans SKN-1/Nrf. *Aging Cell* 8, 524–541.

Steinbaugh MJ, Narasimhan SD, Robida-Stubbs S, Moronetti Mazzeo LE, Dreyfuss JM, Hourihan JM, Raghavan P, Operaña TN, Esmaillie R & Blackwell TK (2015) Lipid-mediated regulation of SKN-1/Nrf in response to germ cell absence. *eLife* 4, e07836.
